# Influence of warming and reanimation conditions on seminiferous tubule morphology, mitochondrial activity, and cell composition of vitrified testicular tissues in the domestic cat model

**DOI:** 10.1371/journal.pone.0207317

**Published:** 2018-11-08

**Authors:** David Baruc Cruvinel Lima, Lúcia Daniel Machado da Silva, Pierre Comizzoli

**Affiliations:** 1 Laboratory of Carnivore Reproduction, School of Veterinary Medicine, State University of Ceará (Universidade Estadual do Ceará, UECE), CEP, Fortaleza, CE, Brazil; 2 Smithsonian Conservation Biology Institute, National Zoological Park, Washington, DC, United States of America; Faculty of Animal Sciences and Food Engineering, University of São Paulo, BRAZIL

## Abstract

Understanding critical roles of warming and reanimation is critical to improve the survival of vitrified testicular tissue in domestic cats. The objective was to study structural and functional properties of testicular tissues from prepubertal domestic cats after standard vitrification followed by two warming protocols (directly at 37°C or with a 5-second pre-exposure to 50°C) and three reanimation time points (immediately, 24 h and 5 days post-warming). In Experiment 1, tissues were evaluated for histo-morphology and mitochondrial activity immediately or 24 h after warming protocols. In Experiment 2, cell viability, DNA fragmentation, and germ cell composition were assessed immediately, 24 h, or 5 days after optimal warming. Preservation of seminiferous tubule structure was better using warming at 50°C for five seconds, and survival of somatic as well as germinal cells was higher compared to direct warming at 37°C for one minute. Short term in vitro culture (for reanimation) also proved that cellular composition and functionality were better preserved when warmed for a short time at 50°C. Collective data showed that short warming at 50°C led to better quality of seminiferous tubule structure and cell composition after vitrification and short-term culture. In addition, data suggest clear directions to further understand and optimize testicular tissue survival after fertility preservation procedures.

## Introduction

Long-term preservation of testicular tissues provides more options to maintain genetic diversity and sustainability in populations of rare and endangered species. Despite other techniques available for pubertal animals (including the rescue of epididymal sperm cells) to subsequently transmit genes to the next generation, testicular tissue is the only biomaterial for preserving the fertility of prepubertal animals that died unexpectedly [1, 2]. In addition, oncological treatments for young boys are gonadotoxic and may lead to infertility [3]. Thus, the cryopreservation of testicular tissue can be an option to preserve the fertility of these cancer patients [4]. Previous studies aiming at establishing optimal protocols to protect biological material for future utilization in assisted reproduction have been conducted in different species such as mouse [5], humans [6], cats [1], dogs [7], and wild species [8]. However, a lot of progress remains to be done in male fertility preservation.

In terms of preservation techniques, vitrification or ultra-rapid freezing is a convenient method [9, 10] that has led to satisfactory results in morphological and structural maintenance of tissues [11, 12]. Cryoprotectant types and exposure, tissue biopsy size, and freezing rates play critical roles in the tissue survival [13, 14]. However, more efforts are needed to understand the influence of warming protocols to (1) prevent devitrification/ice recrystallization in samples and (2) promote optimal reanimation of the tissue [15]. For instance, beneficial effect of short time exposure to high temperatures have already been demonstrated in warmed mouse oocytes; with 80% of survival using than approach compared to 0% at regular temperatures [16]. Few studies have been conducted on males germplasm. Using testicular tissue, different warming protocols after freezing tests from bovine were compared [17]. In this only study reported in the literature, authors evaluated cell viability after thawing and spermatogonia enrichment after 24 h of culture. Warming samples at 37°C for 3 minutes or 97–100°C for 30–40 seconds led in both cases to >85% of cell viability. Warming at 37°C for 1 minute have been used for vitrified testicular tissue in different species, including cats [1, 10]. Although quick exposure to high temperatures is beneficial for oocytes [16], warming conditions using similar approaches have never been evaluated in testicular tissues, including in domestic cats. Furthermore, the use of tissue culture to assess cellular reanimation has not been thoroughly studied in any species. Thus, replacing usual warming at 37°C for vitrified testicular tissue by optmizied conditions can contribute to improve the cryopreservation protocols and tissue reanimation.

Spermatogenesis is a complex process requiring adequate germ cell environment that may be impaired after warming and reanimation [18, 19]. Production of spermatozoa from frozen-thawed testicular tissue (followed by the birth of healthy offspring after oocyte fertilization and embryo transfer) is possible in the mouse model [11, 20]. Structure and functions of seminiferous tubules, including the vimentin filaments in Sertoli cells and connections between germ cells have to be properly preserved [12]. Sertoli cells also have to fully support germ cell development though signaling and metabolic process [21]. Intact communication between cells is critical for a normal spermatogenesis development after cryopreservation. Connexin 43 is the main component of the cellular junctions which is abundant in mammalian testicular tissues [22]. Besides structural properties [20], cellular functions must be preserved, especially for the increase production of energy that is needed after freezing and thawing. Thus, mitochondrial activity should be maintained after cryopreservation and during in vitro culture [23].

Subsequent success of in vitro spermatogenesis (and sperm production) is determined by the quantity of live germ cells after warming [24]. In addition, nuclear DNA must be intact and apoptosis has to be prevented to ensure the fertility preservation [25, 26]. In terms of key mechanisms that have to be preserved, proteins participating in gene regulation may be used to evaluate the ability of pre-meiotic and meiotic cells to survive and maintain characteristics [27]. Only few studies using proteins such as Oct4 and Boule respectively present in pre-meiotic and meiotic cells exist [28, 29]. Furthermore, the use of these critical markers to evaluate germ cell populations have never been reported in cats.

Reports mentioned above using germ cells from mammal models showed that warming condition makes a difference in cellular and tissue quality after cryopreservation, even though results in the literature is still scarce. However, the influence of warming in testicular tissue vitrified has never been evaluated in domestic cats–a relevant model for wild animals and humans [30].

Therefore, the objective of the study was to evaluate structural and functional properties of vitrified testicular tissue from prepubertal domestic cats exposed to two warming conditions (directly at 37°C or with a 5-second pre-exposure to 50°C) and three reanimation time points (immediately, 24 h and 5 days post-warming).

## Materials and methods

### Collection and dissection of testes

Testes from prepubertal male domestic cats (3 to 6 month old) were obtained after routine orchiectomy at local veterinary clinics and transported in phosphate buffered saline (PBS) at 4°C to the laboratory within 6 h of excision. Testicles were washed once with PBS and dissected from surrounding tissues. Then, they were placed in handling medium composed of Hepes-Ham’s F10 medium (Irvine Scientific #99168) supplemented with 1mM pyruvate, 2 mM L-glutamine, 100 IU/mL penicillin, 100 μg/mL streptomycin, 2.5% foetal bovine serum (FBS) and cut in pieces of approximately 1–2 mm^3^ using scalpel blade and forceps. Smithsonian Conservation Biology Institute’s Animal Care and Use Committee granted a waiver of the animal care and use approval because tissues were from routine neutering that otherwise would be discarded.

### Vitrification of testicular tissue

Tissue biopsies were immediately subjected to multiple evaluations (Fresh group; see below) or exposed to cryoprotectants solution composed of dimethylsulphoxide (DMSO) and glycerol (GLY) (Sigma- Aldrich). Specifically, tissue biopsies were threaded onto a 30-G needle (BD Precision Glide needle, Fischer Scientific) [31] and immersed in an equilibrium solution containing 1.4 M of each cryoprotectant, 0.25 M sucrose, and Ham’s F10 for 10 minutes at room temperature (~22°C). Tissues then were exposed to a vitrification solution containing 2.8 M of each cryoprotectant, 0.50 M sucrose, Ham’s F10 and 10% of FBS for 5 minutes at room temperature (~22°C) [1]. After placing threaded tissues on an aseptic absorbent filter to remove the remaining vitrification solution, they were plunged directly into liquid nitrogen, and stored in cryotubes for at least one week.

### Warming of vitrified tissues

Needles with tissue biopsies were immersed in a PBS pre-warmed in a water bath at 37°C for 1 minute or at 50°C for 5 seconds. Needles then were immersed in a solution at room temperature (~22°C) containing decreasing concentrations of sucrose (0.50 M; 0.25 M; 0.00 M), Hepes-Ham’s F10 and 20% of FBS for 5 minutes each in order to remove the cryoprotectants.

### Tissue culture

Warmed tissues were placed into 1cm^2^ pieces of 1.5% agarose gel that were pre-conditioned by immersion in culture medium composed of Hepes-Ham`s F10 (supplemented with 2mM L-glutamine, 1mM pyruvate, 100 IU/ml penicillin, 100 μg/ml streptomycin and 5% FBS). Two tissue biopsies on each gel were incubated in a 4-well culture plate with 400μl of culture medium at 38.5°C in a humidified atmosphere of 5% CO_2_ in air [32]. Samples were either cultured for 24 h or for 5 days with half the volume of culture medium changed every 48 h.

### Histomorphology

Testicular tissues were fixed overnight in Bouin's solution, embedded in paraffin, sectioned in series (5 μm thickness), mounted on slides, and stained with hematoxylin-eosin [12]. Slides were analyzed using a microscope equipped for digital photomicrography (SPOT advanced software 5.0; Diagnostic Instruments).

Seminiferous tubule and cell integrity were evaluated according to criteria established by our colleagues [33]. Intact tubules with no detachment of cells from the basement membrane, no rupture of stroma, no swelling of the lamina propria and normal junctions between cells were considered with a normal structure (score 1). Score 0 was attributed to tubules with changes in any of the previous criteria. A total of 30 randomly selected seminiferous tubules for each animal in each experimental group were classified as normal structure (score 1) or damage structure (score 0) totalizing 150 tubules per group. Percentage of normal seminiferous tubules was calculated relative to the total number of observed tubules.

### Vimentin detection by immuno-histochemistry

Following sample collection, warming and culture, tissues were fixed overnight in 4% paraformaldehyde (PFA). Then, they were processed via routine histology, embedded in paraffin and sectioned in series (5μm thickness). After, dewaxing in Xylene and rehydration in decreasing alcohol baths. The antigen retrieval was performed by submerging the slides in citric acid/EDTA buffer at 95°C water bath for 20 minutes. After cooling down, slides were washed in PBS once and PBST (PBS and 0.1% Triton X-100) twice for 3 minutes each. Tissue sections were saturated with 5% bovine serum albumin (BSA) and 0.5% Triton X-100 in PBS for 1 h at room temperature (~22°C in our laboratory). Samples then were incubated with primary antibody (antivimentin—1:500, Abcam #ab8069) overnight at 4°C in a humidified chamber. A negative control in which the primary antibody was omitted was included in each trial. After extensive washings in PBS and PBST, the samples were incubated in PBS for 1 h at room temperature (~22°C). Then, tissue sections were incubated with secondary antibody (anti-mouse IgG– 1:500, Invitrogen #62–6520) for 1 h at 37°C in darkened container. After new washes in PBS and PBST, tissue sections were counterstained with Hoechst 33342 (1:100, Sigma-Aldrich) in a humidified chamber for 10 minutes at room temperature before mounting with Vectashield medium (Vector laboratories) [34]. Images were captured using an Olympus BX41 epifluorescence microscope (Olympus Corporation) with SPOT advanced software 5.0 (Diagnostic Instruments A total of 30 randomly selected seminiferous tubules in each experimental group were assessment. Percentage of the Sertoli cells positive for vimentin (green staining of intact Sertoli cells) was calculated relative to the total number of Sertoli cells observed in each tubule.

### Connexin expression by Western Blot

Testicular biopsies were frozen at -80°C until processed. Tissues were placed in 1.5mL microcentrifuge tubes containing 200 μl of RIPA buffer (Sigma-Aldrich) supplemented with protease inhibitor mix (Sigma-Aldrich) and homogenized with sonicator on ice. Homogenate then was centrifuged at 14,000 x g for 20 minutes at 4°C. Supernatant was removed and used for measuring the total protein concentration using the BCA Protein Assay Kit (Thermo Scientific). Samples were diluted with 2x Laemmli Sample Buffer (Bio-Rad) and proteins were denatured at 95°C for 10 minutes. For electrophoresis, 10mg of proteins from each experimental group was placed in well of 4–15% polyacrylamide gels (Bio-Rad) and proteins were separated in runner buffer at 95V for 1 h. Proteins then were transferred onto nitrocellulose membranes in transfer buffer at 100V for 1 h. Membranes were placed in a blocking solution containing Tris-buffered saline in 0.1% Triton X-100 (TBST) and 5% BSA at room temperature for 1 h. Membranes then were incubated with primary antibody connexin 43 (1:500, Cell Signaling #3512) or β-tubulin (1:400, Thermo Scientific #MA5-11732) that was used as loading control overnight at 4°C. Membranes were rinsed in TBST for 10 minutes three times and incubated with secondary antibody anti-rabbit (1:3000, Invitrogen #65–6120) to connexin 43 or anti-mouse (1:5000, Invitrogen #626520) to β-tubulin at room temperature for 1 h. Proteins bands were detected with Clarity Western ECL Substrate (Bio-Rad) and membranes were exposed in a ChemiDoc XRS imaging system (Bio-Rad) [34]. Intensity signals of proteins were calculated using ImageJ version 1.51 software (ImageJ; National Institute of Health, Bethesda, MD, USA). Measured area was the same for all proteins bands. Connexin and tubulin optical densities were determined by subtracting background from protein signals in each sample. Final optical density was obtained subtracting the connexin density from the tubulin density. The optical density of Connexin 43 was analyzed relative to intensity of β-tubulin in all experimental groups.

### Mitochondrial membrane potential

Tissues were incubated in a darkened container with 10μg/mL Rhodamine 123 (Invitrogen) in Ham`s F10 medium supplemented with 10% FBS for 15 minutes at 37°C. Tissues then were washed in PBS twice for 1 minute each and were mounted on slides with Vectashield mounting medium (Vector laboratories). Images were captured using an Olympus BX41 epifluorescence microscope (Olympus Corporation) with SPOT advanced software 5.0 (Diagnostic Instruments) at ×100. Parameters of the microscope camera settings were the same throughout the experiments. Pixel intensity of Rhodamine 123 (mean grey value) was determined using ImageJ version 1.51 software (ImageJ; National Institute of Health, Bethesda, MD, USA). Measured area was the same for all samples and the relative intensity was determined by subtracting the tissue Rhodamine 123 fluorescence emission of the background of each slide. We evaluated 30 randomly selected areas in each experimental group and high mitochondrial membrane potential was considered the tissue with high intensity of Rhodamine 123 [35].

### ATP concentration

Fifteen mg of tissue were washed in cold PBS twice. Tissues were homogenized in 100 μL of ATP Assay Buffer with a sonicator. Resulting suspension then was centrifuged for 4 minutes at 4°C at 13,000 x g and supernatant was transferred to a new tube. Samples were deproteinized and neutralized using the Deproteinizing Sample Preparation Kit–TCA (Abcam #ab204708). ATP concentration was measured using ATP assay kit (Abcam #ab 83355). Briefly, the ATP Reaction Mix was prepared and added to all standards and samples in a 96 well plate. Plates were incubated in a darkened container for 30 minutes at room temperature. Fluorescence was read using a microplate reader (SpectraMax Gemini XPS, Molecular Devices) with the excitation/emission setting at 485/590nm. The ATP concentration in the samples was calculated relative to the standard curve.

### Viability and DNA fragmentation

We used the In-Situ Cell Death Detection kit (Roche) following the manufacturer’s instructions. Cells present in tissue biopsies from all groups were extracted by slicing with a scapel blade in Modified Ham`s F-10 Basal Medium–HEPES (Irvine Scientific) supplemented with 2mM L-glutamine, 1mM pyruvate, 100 IU/mL penicillin, 100 μg/mL streptomycin and 5% FBS. Cells suspensions were centrifuged at 300 × g for 8 minutes and resuspended in fresh Ham`s F10 medium (1:1). After, 20 μL of suspension was smeared on a glass slide. The cells were fixed with 4% paraformaldehyde for 1 h at room temperature and permeabilized with 0.1% Triton X-100 in 0.1% sodium citrate in PBS (PBS-T) on ice for 2 minutes. The TUNEL reaction mixture was prepared using the enzyme solution composed by terminal deoxynucleotidyl transferase (TdT) and label solution composed by nucleotide polymers. The slides were rinsed twice with PBS and incubated with TUNEL reaction mixture for 1 h at 37°C within a humidified darkened container. A negative control in which the TdT was omitted was included in each trial. The positive control was performed incubating the cells with DNase I recombinant (Sigma-Aldrich) for 10 minutes before to labeling procedures to induce DNA stands breaks. The nucleus of all cells was stained with Hoechst 33342 (1:100, Sigma-Aldrich) and the nucleus of dead cells were counterstained with propidium iodide (1:100, Invitrogen) in a humidified chamber for 10 minutes at room temperature and then, the slides were mounted with Vectashield mounting medium (Vector laboratories). Fragments from fresh tissue were cultured for 24h and 5 days such as control to evaluate the warming influence in survival and cell apoptosis after culture. We evaluated 1,000 cells per experimental group and the cell viability was calculated considering the total viable cells (normal DNA integrity and fragmented DNA) in relation to dead cells. Proportion of cells with fragmented DNA was calculated in relation to the total number of live cells. Cells with positive DNA fragmentation showed a bright green nucleus and dead cells showed a bright red nucleus [26]. Images were captured using an Olympus BX41 epifluorescence microscope (Olympus Corporation) with SPOT advanced software 5.0 (Diagnostic Instruments).

### Germ cell identification

Using specific markers for pre-meiotic and meiotic cells, we evaluated the germ cell progression in testicular tissue from prepubertal cats. Testicular cells were isolated by slicing with a scapel blade in Modified Ham`s F-10 Basal Medium–HEPES (Irvine Scientific) supplemented with 2mM L-glutamine, 1mM pyruvate, 100 IU/ml penicillin, 100 μg/ml streptomycin and 5% FBS. Cells suspensions were centrifuged at 300 × g for 8 minutes and resuspended in fresh Ham`s F10 medium (1:1). After, 20μl of suspension was smeared on a glass slide. The cells were fixed with 4% paraformaldehyde for 1 h at room temperature and permeabilized with 0.1% Triton X-100 in PBS (PBS-T) for 3 minutes. Cells were saturated in 5% BSA in PBS for 1 h at room temperature and incubated with the pre-meiotic marker anti-OCT4 (1:200, Abcam #ab137427) or the meiotic marker anti-BOULE (1:100, Abcam #ab28745) overnight at 4°C in a humidified chamber. Slides were washed in PBS twice and PBS-T once for 5 minutes each and then incubated with secondary antibodies (donkey anti-goat IgG-FITC, Santa Cruz Biotechnology #2024; 1:100 goat anti-rabbit IgG-TR, Santa Cruz Biotechnology #2780) for 1 h at 37°C in darkened container. The nucleus was stained with Hoechst 33342 (1:100, Sigma-Aldrich) in a humidified chamber for 10 minutes at room temperature and then, the slides were mounted with Vectashield medium (Vector laboratories) [36].

We evaluated 500 cells in duplicate per experimental group and calculated the proportion of cells with positive staining for both antibodies. Images were captured using an Olympus BX41 epifluorescence microscope (Olympus Corporation) with SPOT advanced software 5.0 (Diagnostic Instruments).

### Experimental design and statistical analysis

For each experiment, fresh tissue had not been exposed to vitrification and any warming process was used a control. For each of the described experiments, tissues were randomly allocated to different treatments.

Experiment 1 was conducted to evaluate the influence of warming on tissue structure and mitochondrial activity (n = 6 replicates). For each replicate, testicular tissue biopsies were collected from 14 testicular pairs and allocated to control group (fresh tissue) or vitrified. After storage for a minimum of 1 week in liquid nitrogen, samples were warmed at 37°C for 1 minute in PBS (Warming 1) followed by in vitro culture for 24h (Culture 1) or warmed at 50°C for 5 seconds in PBS (Warming 2) followed by in vitro culture for 24h (Culture 2). For histomorphological evaluation of each replicate, 1–2 biopsies from 8 testicular pairs were fixed in Bouin's solution for all experimental groups. For vimentin assessment, 1–2 testicular pieces from 8 testicular pairs were fixed in 4% PFA for all experimental groups. For connexin evaluation, 1 testicular piece from 6 testicular pairs was frozen at -80°C for all experimental groups. For mitochondrial membrane potential evaluation, 1–2 testicular pieces from 6 testicular pairs were incubated with Rhodamine 123 to all experimental groups and 1 testicular piece from 6 testicular pairs was used to measure the ATP concentration in all experimental groups.

Experiment 2 was based on results of Experiment 1 to further evaluate the influence of optimal warming on tissue survival and germ cell composition (10 replicates) after short in vitro culture periods (samples were warmed with the protocol at 50°C only and an extra evaluation after 5 days of culture was added). For each replicate, testicular tissue biopsies were collected from testicular pairs and allocated in control group (fresh tissue) or submitted to vitrification process. After storage for a minimum of 1 week in liquid nitrogen, samples were warmed at 50°C for 5 seconds followed by in vitro culture for 24h and 5 days. For Viability, DNA fragmentation, Boule and Oct4 assessment, 1–2 testicular pieces from 5 testicular pairs were homogenized and smeared on glass slide for all groups.

Data were expressed as mean and standard error and analyzed using the statistical software graphpad prism version 5.01 (GraphPad Software Inc., San Diego, CA, USA). Data distribution was tested with the Shapiro-Wilk test. To compare average proportions between groups with normal distribution, T test or analysis of variance (ANOVA) followed by Tukey test was used. If data distribution was not normal Wilcoxon test (non-parametrical test) was used to compare values. Differences were considered significant when P < 0.05.

## Results

### Experiment 1: Influence of warming condition on seminiferous tubule morphology and structure

**Fig 1:**
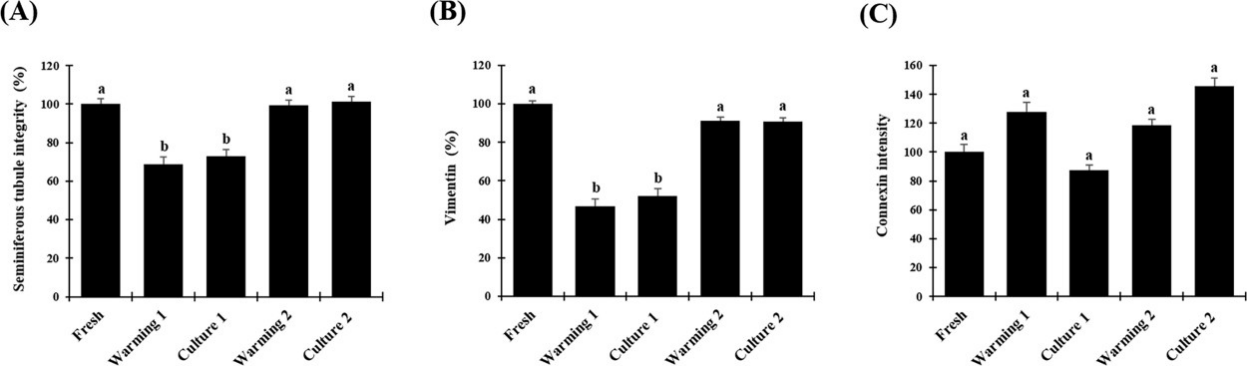
Morphological evaluation of vitrified testicular tissue from prepubertal domestic cats. (A) proportion of intact seminiferous tubules, (B) proportion of Sertoli cells stained with vimentin in each seminiferous tubule, (C) connexin expression relative to pixel intensity. Data are expressed as mean ± SE (n = 6 animals per treatment). Different letters above bars indicate significant statistical differences between treatments (P < 0.05). Warming 1 (tissue warmed at 37°C), culture 1 (warming 1 followed by culture for 24h), warming 2 (tissue warmed at 50°C), culture 2 (warming 2 followed by culture for 24h).

**Fig 2:**
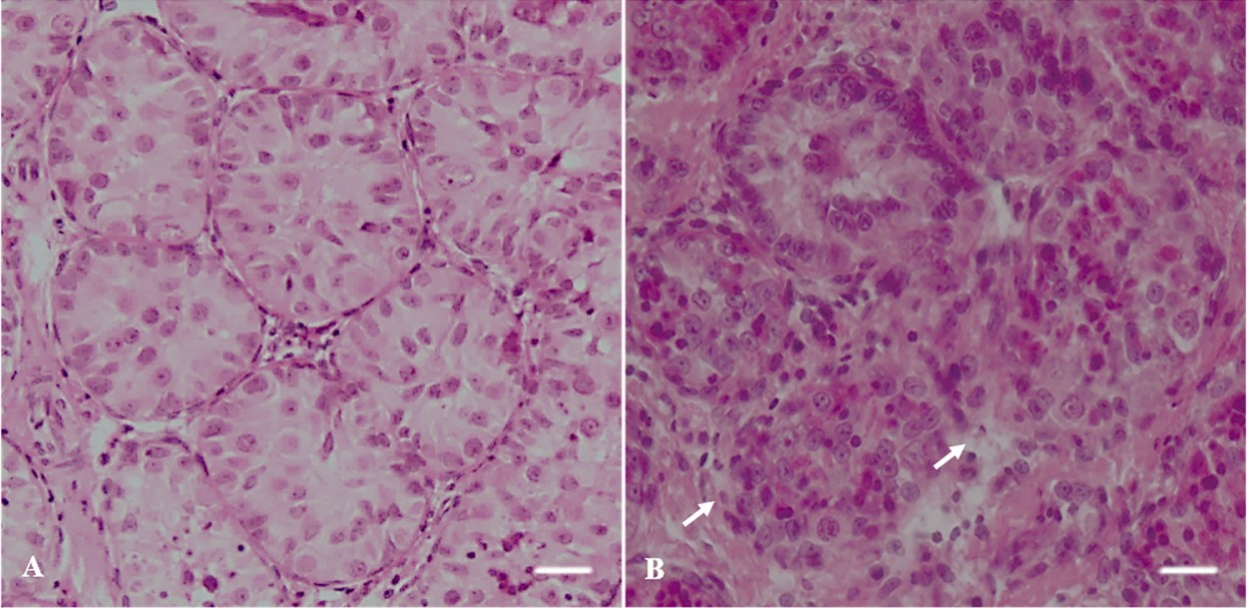
Histological evaluation of seminiferous tubules in testicular tissue from prepubertal cats. (A) seminiferous tubules with intact morphology (Score 1; HE), (B) seminiferous tubules with degenerated morphology (Score 0; HE). White arrows indicate degenerated seminiferous tubule. Bar = 5 μm.

Average percentages of intact seminiferous tubules were 100.0% ± 2.8 in fresh tissues but were lower (P < 0.05) after warming at 37°C (68.9% ± 3.7) and after in vitro culture for 24 h (72.9% ± 3.6; Figs 1A and 2). However, proportions of intact tubules after warming at 50°C (99.3% ± 2.8) and in vitro culture (101.3% ± 2.7) were higher (P < 0.05) than the other treatment group and remained similar to the fresh control (P > 0.05; Figs 1A and 2).

**Fig 3:**
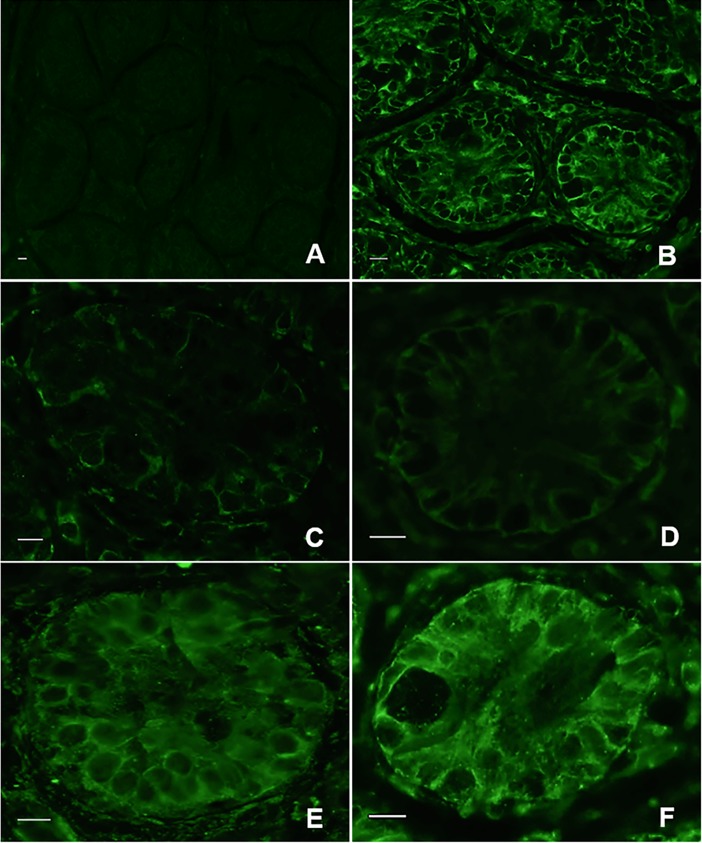
Vimentin in testicular tissue from prepubertal cats. (A) negative control, (B) fresh tissue, (C) Warming 1 (tissue warmed at 37°C), (D) culture 1 (warming 1 followed by culture for 24h), (E) warming 2 (tissue warmed at 50°C), (F) culture 2 (warming 2 followed by culture for 24h). Bar = 0.5μm.

Average percentages of tubules with vimentin were 100.0% ± 1.7 in fresh tissues but were lower (P < 0.05) after warming at 37°C (46.8% ± 3.7) and after in vitro culture for 24 h (52.2% ± 3.9; Figs 1B and 3). However, proportions of Sertoli cells stained with vimentin after warming at 50°C (91.3% ± 1.8) and in vitro culture (90.7% ± 2.0) were higher (P < 0.05) than the other treatment group and remained similar to the fresh control (P < 0.05; Figs 1B and 3).

**Fig 4:**
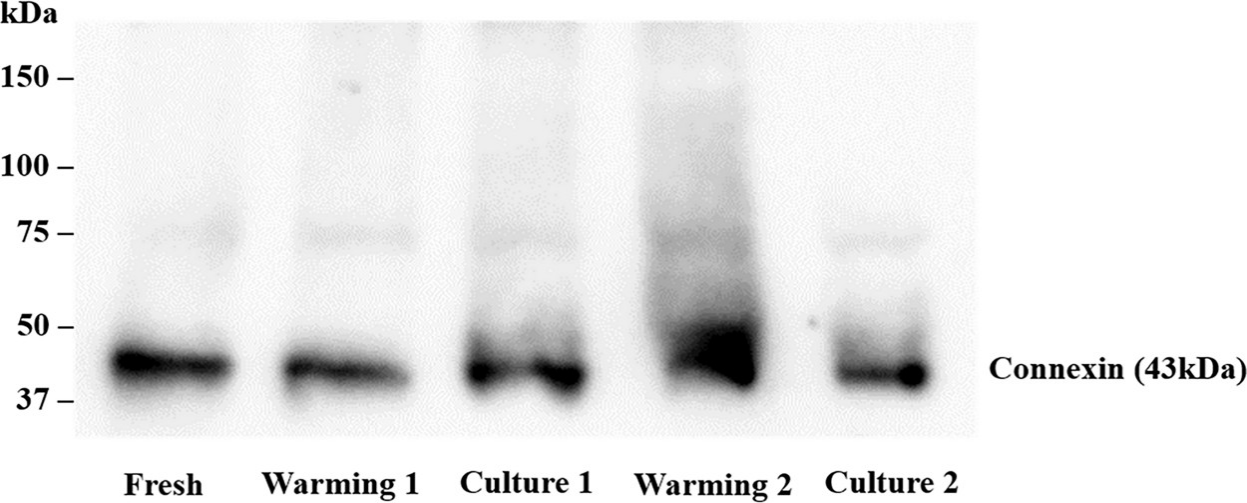
Connexin expression on testicular tissue from prepubertal cats. Warming 1 (tissue warmed at 37°C), culture 1 (warming 1 followed by culture for 24h), warming 2 (tissue warmed at 50°C), culture 2 (warming 2 followed by culture for 24h).

Intensity of Connexin 43 expression in fresh tissue was set at 100.0 ± 5.2. Relative intensity was not different (P > 0.05) in tissues warmed at 37°C (127.9 ± 6.3) and then in vitro cultured (87.3 ± 3.9) or warmed at 50°C (118.6 ± 3.9) before in vitro culture (145.7 ± 5.6; Figs 1C and 4).

### Experiment 1: Influence of warming condition on the tissue metabolism

**Fig 5:**
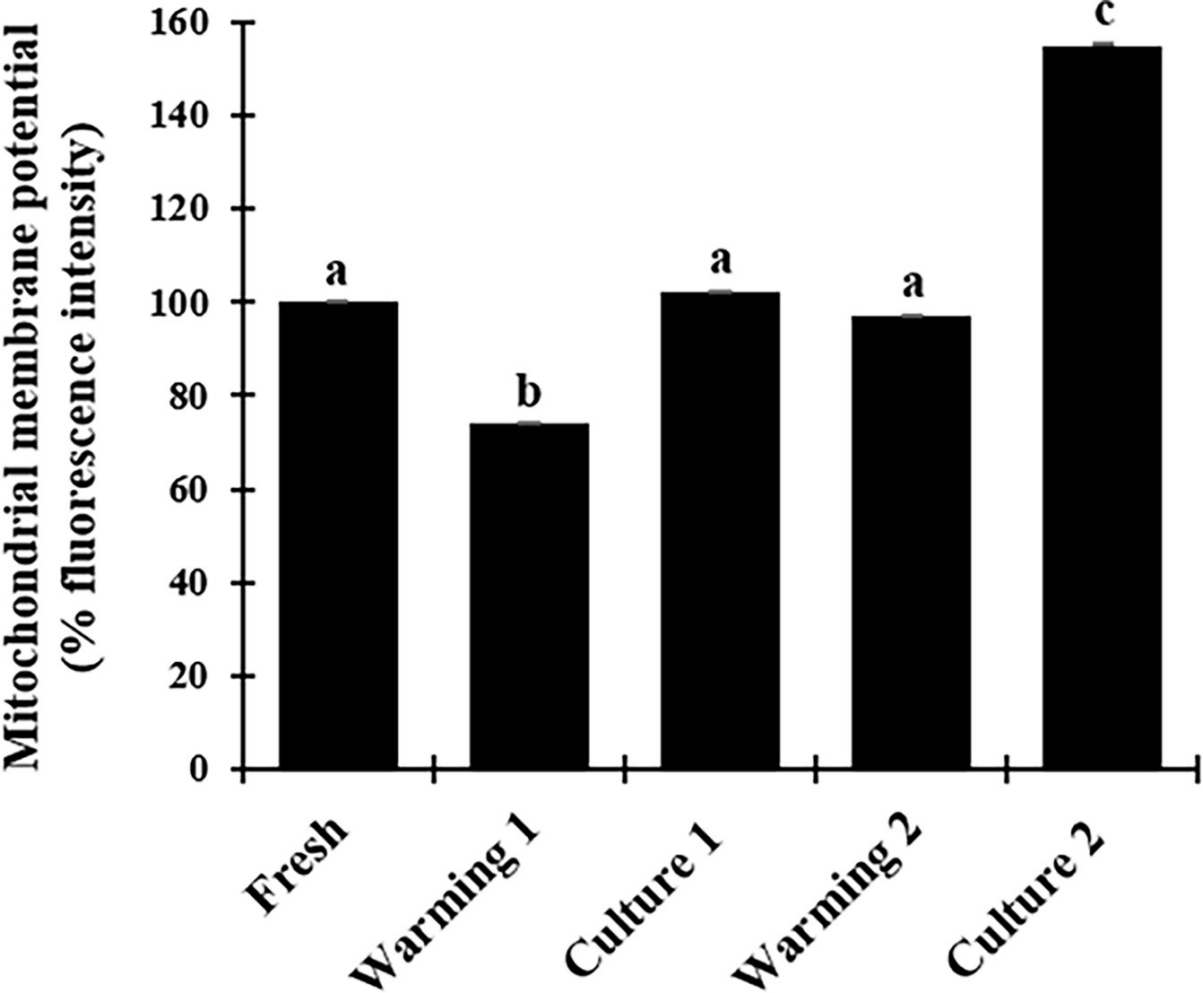
Fluorescence intensity of mitochondrial membrane potential in testicular tissue from prepubertal cats. Data are expressed as mean ± SE (n = 6 animals per treatment). Warming 1 (tissue warmed at 37°C), culture 1 (warming 1 followed by culture for 24h), warming 2 (tissue warmed at 50°C), culture 2 (warming 2 followed by culture for 24h). Different letters above bars indicate significant statistical differences between treatments (P < 0.05).

**Fig 6:**
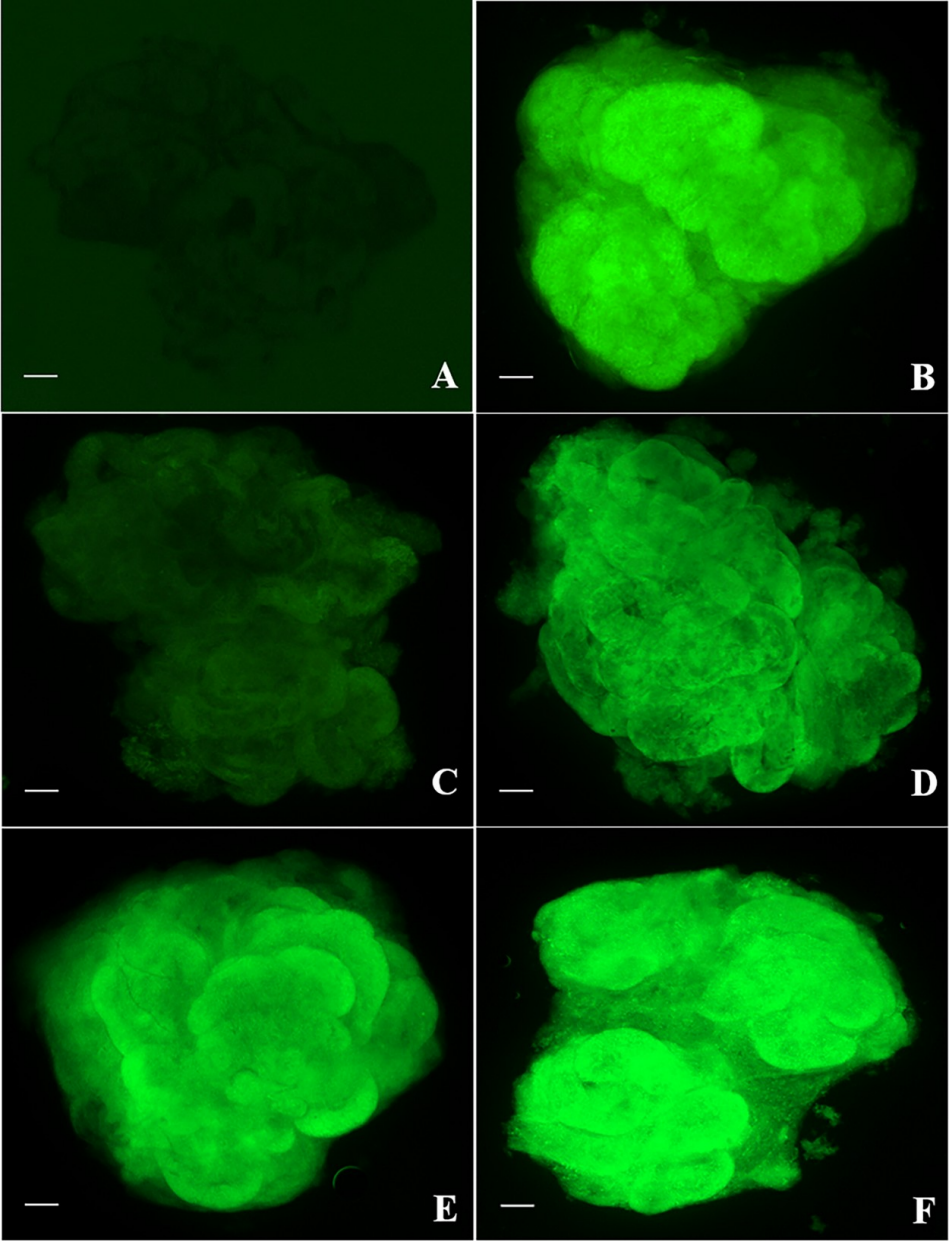
Fluorescence of Rhodamine 123 in testicular tissue from prepubertal cats. (A) negative control, (B) fresh tissue, (C) Warming 1 (tissue warmed at 37°C), (D) culture 1 (warming 1 followed by culture for 24h), (E) warming 2 (warmed at 50°C), (F) culture 2 (warming 2 followed by culture for 24h). Bar = 2 μm.

The fluorescence intensity related to mitochondrial membrane potential was set at 100.0 ± 0.3. Relative intensity was lower (P < 0.05) after tissue were warmed at 37°C (74.0 ± 0.3) but not different (P > 0.05) following in vitro culture (102.0% ± 0.2; Figs 5 and 6). Relative intensity did not vary (P > 0.05) after warming at 50°C (97.0% ± 0.2) but increased after culture (155.0% ± 0.1) and was significantly higher (P < 0.05) than other groups (Figs 5 and 6).

To further understand the variations of mitochondrial membrane potential, ATP production was measured in the same treatment groups but no difference was detected (P > 0.05). Values ranged from 0.13 nmol/μl to 0.27 nmol/μl.

### Experiment 2: Influence of warming condition on testicular cell survival

**Fig 7:**
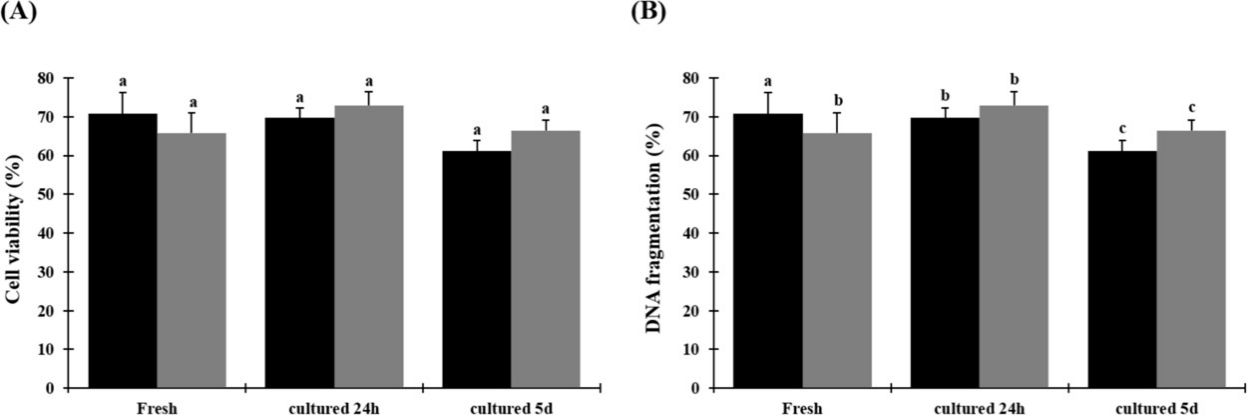
Cell viability and DNA fragmentation from prepubertal cats after warming, 24 h, or 5 days of in vitro culture. (A) cell viability, (B) cells with fragmented DNA. Data are expressed as mean ± SE (n = 5 animals per treatment). Black bars indicate fresh tissue and grey bars indicate vitrified tissue. Different letters above bars indicate significant statistical differences between treatments (P < 0.05).

**Fig 8:**
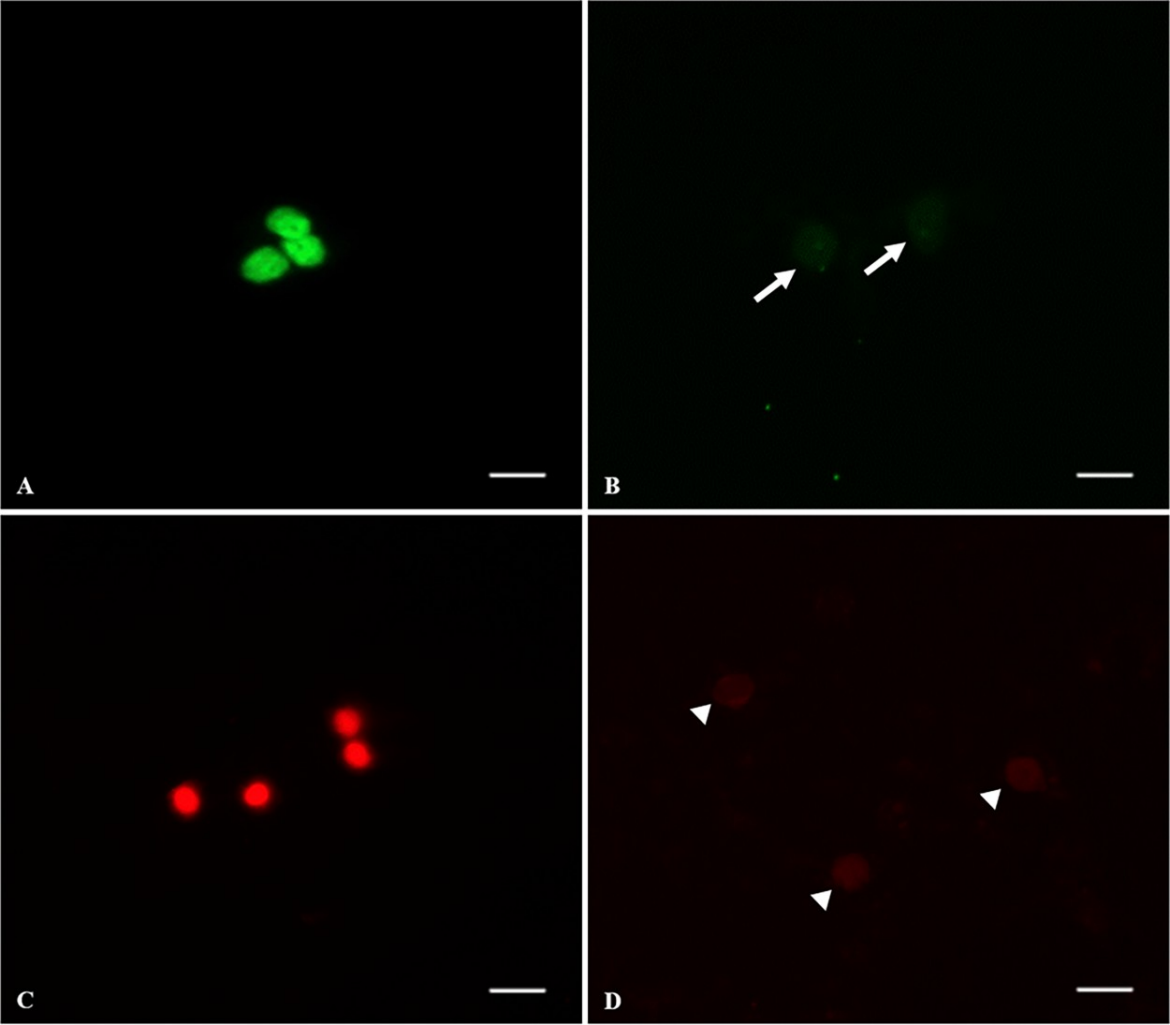
Representative dead cells and fragmented DNA on testicular tissue from prepubertal cats after warming, 24 h, and 5 days of in vitro culture. (A) live cells with fragmented DNA showing bright green nucleus, (B) live cells with intact membrane (white arrows), (C) dead cells showing bright red nucleus, (D) live cells (white arrow heads). Bar = 5 μm.

Based on results of Experiment 1, one warming condition (short exposure to 50°C) was further investigated. Percentages of viable cells did not differ between fresh tissues (70.7 ± 5.4), warmed (65.7 ± 5.4) and culture for 24 h (72.8 ± 3.7) or 5 days (66.3 ± 2.8; P > 0.05) (Figs 7A and 8). Percentages of cells with fragmented DNA after warming (41.4% ± 2.3) and in vitro culture for 24 h (34.9% ± 5.4) and 5 days (62.4% ± 4.3) were significantly higher (P < 0.05) than in the fresh group (17.6% ± 2.8). Percentages did no vary (P > 0.05) between groups after warming and in vitro culture for 24 h and both equally increased after culture for 5 days (P > 0.05) (Figs 7B and 8).

### Experiment 2: Influence of warming condition on the germ cell composition

**Fig 9:**
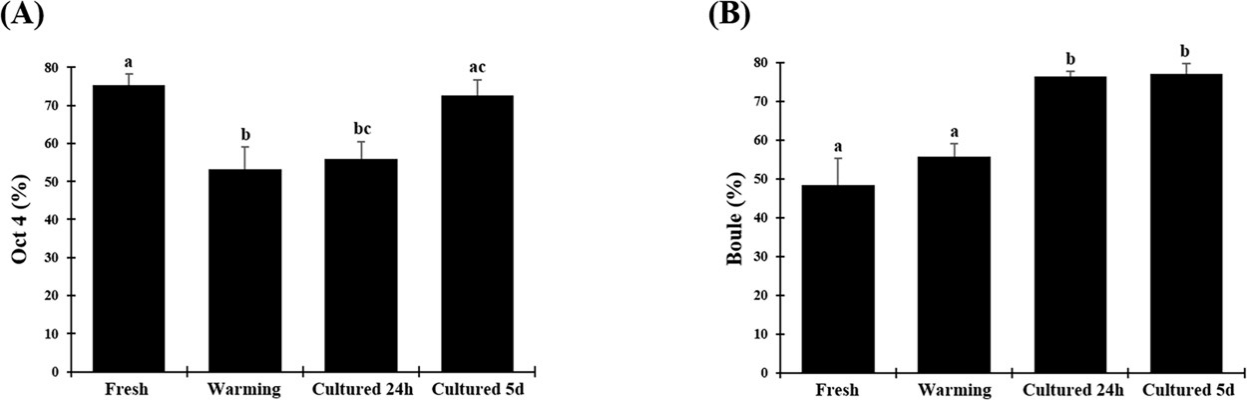
Evaluation of germ cell progression on testicular tissue from prepubertal cats after vitrification and culture using specific markers. (A) Percentage of cells labelled with Oct4 (spermatogonia, pre-meiotic stage), (B) Percentage of cells labelled with Boule (spermatocytes, meiotic stage). Data are expressed as mean ± SE (n = 5 animals per treatment). Different letters above bars indicate significant statistical differences between treatments (P < 0.05).

**Fig 10:**
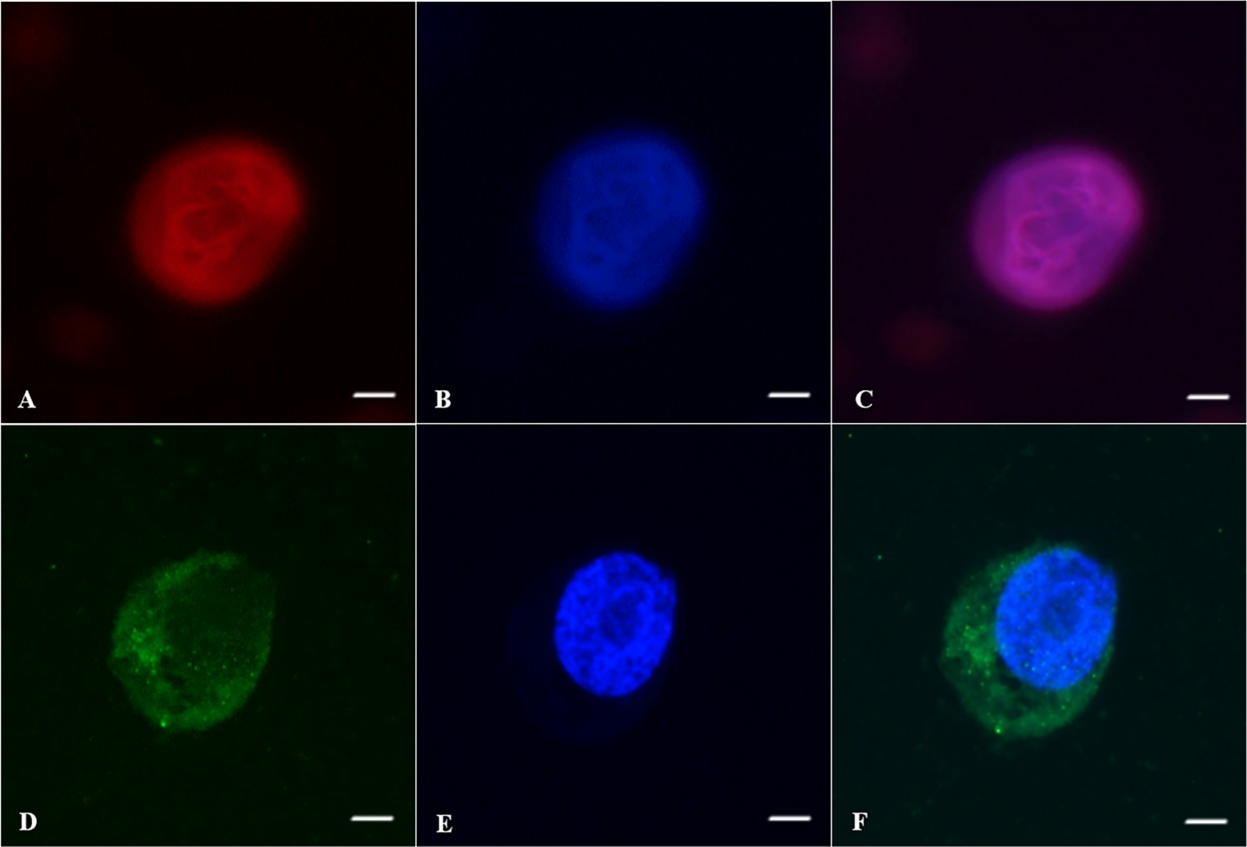
Oct4 and Boule in male germ cells from prepubertal cats after vitrification and culture. (A) Oct4 expression in the nucleus of pre-meiotic cell, (B) nucleus of pre-meiotic cell stained with Hoechst, (C) pre-meiotic cell merged images, (D) Boule expression in the cytoplasm of meiotic cell, (E) nucleus of meiotic cell stained with Hoechst, (F) meiotic cell merged images. Bar = 5 μm.

Percentages of germ cells labelled with Oct4 after warming (53.3% ± 5.8) and after in vitro culture for 24 h (55.9% ± 4.6) were lower (P < 0.05) than in the fresh group (75.3% ± 3.0). However, percentages after in vitro culture for 5 days (72.7% ± 4.0) did not differ between warmed and fresh group (P > 0.05) (Figs 9A and 10).

Percentages of germ cells labeled with Boule were similar (P > 0.05) in fresh tissues (48.4% ± 6.9) and after warming (55.8% ± 3.4). After culture for 24 h (76.5% ± 1.3) or 5 days (77.0 ± 2.9), percentages were higher (P < 0.05) than in the other groups (Figs 9B and 10).

## Discussion

Rapid warming enhanced survival and reanimation of vitrified prepubertal testicular tissue from prepubertal domestic cats. Specifically, structural and functional properties were positively impacted by short exposure to high temperature during warming. We also demonstrated that germ cell composition and differentiation were not altered after vitrification and proper warming [37, 38].

Structural integrity of the testicular tissue is essential for germ cells to develop after cryopreservation treatments. The prevention of ice recrystallization during warming proceedings is essential to preserve the quality of biological components present on tissues and warming rates are involved in this process [15,16]. In our study, higher warming rate for a short time exposure preceded the ice recrystallization, which allowed a better structural quality after warming and provided necessary conditions for tissue reanimation. Previous studies using domestic and wild mammals demonstrated that different aspects may influence the morphology of the seminiferous tubules such as separation and shrinkage of cells from basal membrane [1, 39, 40]. In testicular tissue with compromised morphology the survival and proliferating ability of cells decreased [12, 41]. Our study in the prepubertal cat model throroughly evaluated the influence of different warming conditions on the seminiferous tubules. It was interesting to note that warming condition influenced the quantity of intact seminiferous tubules after in vitro culture and that a short culture period (24 h) was necessary for recovery. In addition, the prevalence of vimentin (a major component of the Sertoli cells cytoskeleton [42]) in the tubules enabled us to verify that critical cells supporting spermatogenesis were preserved [43]. A former study compared fresh and frozen-thawed testicular tissues cultured for 24 h from prepubertal boys. No structural changes were observed in the Sertoli cells staining with vimentin marker [44]. In our study, similar results were observed after warming at 50°C and culture for 24 h. However, after warming at 37°C and culture for 24 h the vimentin presence in Sertoli cells decreased significantly, which evidence the sensibility of these cells to warming [45].

Gap junctions are involved in fundamental intercellular communication during spermatogenesis, being constituted of different transmembrane proteins—connexins [46], with Connexin 43 being the most common connexin in testicular tissue. These proteins are involved with spermatogenic regulation and fertility [47]. In the present study, protein quantity did not change after different warming conditions, which is a good indicator suggesting that these proteins are more resilient than vimentin.

In terms of cell metabolism, the vitrification induces damages in the germ cells, resulting in a loss mitochondrial activity. These organelles are sensitive to non-physiological conditions and modifications in theirs structures increase the reactive oxygen species levels culminating with apoptotic cell death [48]. While other studies in different species have evaluated sperm mitochondrial membrane potential [49–51], our study in the cat was the first to investigate the ability of germ cells from prepubertal animals to recover their mitochondrial potential after vitrification-warming process in the testicular tissue. Results clearly showed that the cell have the ability to recovery to vitrification procedures after warming at 50°C with consequent increase in mitochondrial activity after short term culture. Similar result in the mitochondrial activity after warming and culture was found in a study with vitrification of ovine oocyte [52]. However, in other research evaluating the mitochondrial membrane potential in frozen-thawed ovaries from domestic cats, their results showed that all treatments groups were lower than fresh group [35]. Cellular energy production is performed in the mitochondria. Thus, to better understanding of these mitochondrial membrane potential variations we measured the ATP production of germ cells from all treatment groups. However, no significantly differences were detected, which support the other metabolism factors are involved in the complex mitochondrial membrane potential activity [23, 53].

During warming tissue, germ cells are exposed to toxic and stressful situations such as contact with cryoprotectant and osmotic shock, which decrease the cell viability [12]. A higher percentage of dead cells after warming were observed in previous studies using cryopreserved immature testicular tissue [41, 54]. Thus, optimized cryopreservation protocols are necessary to maintain a higher percentage of live cells after cryopreservation. In this perspective, the adequate time and temperature for tissue to be exposed during warming condition is essential to minimize cell death [55].

Furthermore, ideal culture conditions to maintain the live cells with structural and functional integrity are not yet established [56]. Thus, these cells may suffer injuries during culture, such as DNA fragmentation [57]. Our results showed a higher percentage of live cells after warming and culture. However, the percentage of cells with fragmented DNA increased after warming and culture. Despite these findings the vitrified tissue presented similar degeneration in comparison to cultured fresh tissue, which means that vitrification and warming conditions did not alter cell DNA structure.

In this perspective, the main objective of sustaining live cells during culture is to promote the progression of these cells to advanced stages. Thus, spermatozoa obtained in the culture can be used to artificial insemination [20]. In vitro cell progress of immature testicular tissue was observed in a mouse model [11]. However, spermatozoids obtained from testicular tissue of prepubertal cats has never been reported. Furthermore, the spermatogenesis is a complex process to be understanding, including the participation of different testicular cells and your interactions on this process [58]. In addition, the fundamental mechanisms involved in the reproductive biology, including the in vitro progression of germ cells need to be established [59]. Specific markers previously found in pre-meiotic and meiotic cells such Oct4 and Boule proteins respectively can contribute for improve the comprehension of vitrified germ cell reanimation in vitro culture [37, 38].

The present study was the first to investigate the vitrified-warming germ cell composition and survival using prepubertal testicular tissue from domestic cats. It was particularly interesting to note that after warming the labeled cells decrease, but after short time culture, the pool of pre-meiotic germ cells returned to the same conditions of the fresh tissue. Furthermore, the meiotic cells increased in comparison to the fresh group after 24 h and maintained high percentage after 5 days. In cats, the in vivo spermatogenesis occur approximately at 35°C [11]. However, we used a culture system at 38.5°C to evaluate the cell composition on in vitro culture and it was interesting for us to found proliferation indicative on that temperature. Further studies will be necessary to confirm that the germ cell progression is a continue process after long-term in vitro culture. Furthermore, the benefits of additional components in the testicular culture medium should be investigated. This knowledge will provide new advances for in vitro cell differentiation, which consequently advances in endangered species conservation and human reproductive medicine [30].

In conclusion, warming at 50°C for five seconds can be successfully used to ensure reanimation and survival of vitrified testicular tissues from prepubertal domestic cats. Collective data suggest clear directions to further understand testicular tissue survival after exposure to non-physiological conditions for fertility preservation purpose.
